# Ubiquitous regulation of cerebrovascular diseases by ubiquitin‐modifying enzymes

**DOI:** 10.1002/ctm2.1719

**Published:** 2024-05-22

**Authors:** Jingyong Huang, Zhenhu Zhu, Dirk Schlüter, Kate Lykke Lambertsen, Weihong Song, Xu Wang

**Affiliations:** ^1^ Department of Vascular Surgery The First Affiliated Hospital of Wenzhou Medical University Wenzhou China; ^2^ School of Pharmaceutical Sciences Wenzhou Medical University Wenzhou China; ^3^ Institute of Medical Microbiology and Hospital Epidemiology, Hannover Medical School Hannover Germany; ^4^ Department of Neurobiology Research Institute of Molecular Medicine University of Southern Denmark Odense C Denmark; ^5^ BRIGDE—Brain Research—Inter‐Disciplinary Guided Excellence, Department of Clinical Research University of Southern Denmark Odense C Denmark; ^6^ Department of Neurology Odense University Hospital Odense C Denmark; ^7^ Oujiang Laboratory Key Laboratory of Alzheimer's Disease of Zhejiang Province Zhejiang Provincial Clinical Research Center for Mental Disorders Institute of Aging School of Mental Health Affiliated Kangning Hospital The Second Affiliated Hospital Yuying Children's Hospital Wenzhou Medical University Wenzhou China

**Keywords:** cerebrovascular disease, deubiquitinating enzyme, disease mechanism, therapeutic target, ubiquitinating enzyme, ubiquitination

## Abstract

Cerebrovascular diseases (CVDs) are a major threat to global health. Elucidation of the molecular mechanisms underlying the pathology of CVDs is critical for the development of efficacious preventative and therapeutic approaches. Accumulating studies have highlighted the significance of ubiquitin‐modifying enzymes (UMEs) in the regulation of CVDs. UMEs are a group of enzymes that orchestrate ubiquitination, a post‐translational modification tightly involved in CVDs. Functionally, UMEs regulate multiple pathological processes in ischemic and hemorrhagic stroke, moyamoya disease, and atherosclerosis. Considering the important roles of UMEs in CVDs, they may become novel druggable targets for these diseases. Besides, techniques applying UMEs, such as proteolysis‐targeting chimera and deubiquitinase‐targeting chimera, may also revolutionize the therapy of CVDs in the future.

## Graphical abstract

1. Ubiquitin‐modifying enzyme (UMEs), comprising E1, E2, E3 ubiquitinating enzymes and deubiquitinating enzymes, orchestrate ubiquitination and thereby critically regulate the pathophysiology of cerebrovascular diseases (CVDs).
2. Alteration in the abundance or activity of UMEs affects the outcome of CVDs.
3. UME‐targeting therapy and therapeutic techniques applying UMEs may be beneficial for the treatment of CVDs.

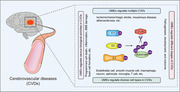

## 1 INTRODUCTION

As the “central processing unit” of the body, the brain needs a relatively large amount of energy to maintain its sophisticated functionality. Although its weight accounts for only 2% of the body weight, the brain consumes about 20% of the human body's oxygen and glucose supply.^1^, ^2^ Unlike other major energy consumers of the body, such as the liver, the brain has barely any reservation of energy materials. Therefore, the cerebrovascular system is the only important source of glucose and oxygen for the brain. Cerebral blood flow, which is mainly supplied by internal carotid arteries and vertebral arteries, accounts for up to 20% of total cardiac output.^3^, ^4^ Given the importance of the cerebrovascular system in brain energy supply, interruption of the cerebral blood supply leads to termination of cerebral electrical activity and irreversible brain damage within minutes. Chronic cerebrovascular diseases (CVDs), such as moyamoya disease (MMD) and intracranial atherosclerotic disease, cause insufficient blood supply to the brain, inciting symptoms including headache, visual disturbance, paresthesia, motor nerve dysfunction, mental abnormality, and cognitive impairment.^5^, ^6^, ^7^, ^8^, ^9^ These chronic CVDs and other factors, for example, hypertension, can also trigger life‐threatening acute cerebrovascular accidents such as ischemic and hemorrhagic stroke.^5^, ^6^, ^8^ CVDs have become the main cause of disability and mortality in both developing and developed countries.^10^

**FIGURE 1:**
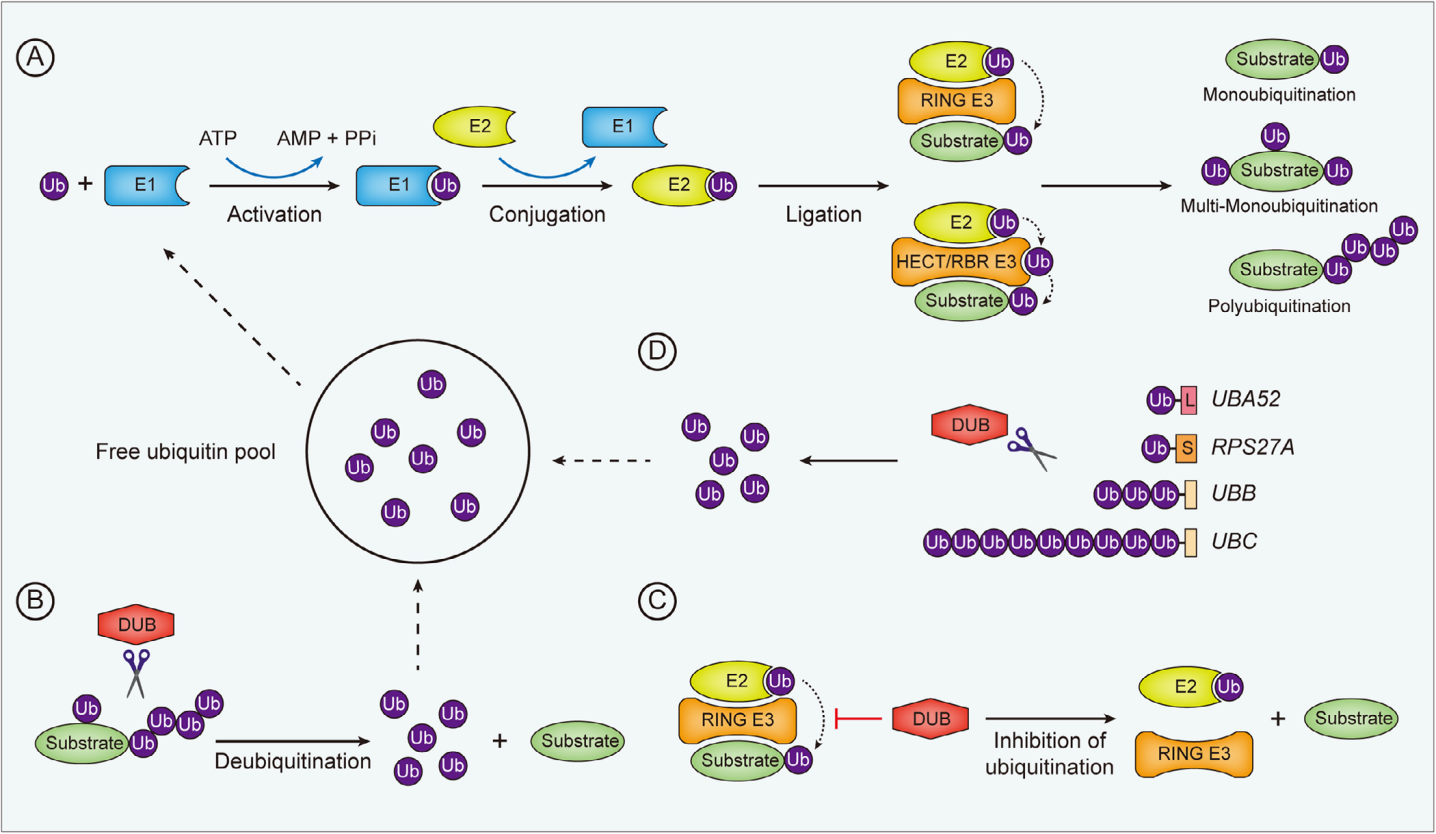
Overview of the process of ubiquitination and deubiquitination. (A) Ubiquitination is an enzymatic cascade catalyzed sequentially by E1s, E2s, and E3s. Ubiquitin is first activated by an E1 and subsequently transferred to an E2. An E3 mediates the final attachment of the ubiquitin molecule to a protein substrate. In contrast to HECT and RBR E3s, which receive ubiquitin molecules from E2s before transferring to substrates, RING E3s mediate the transfer of ubiquitins directly from E2s to substrates. (B,C) Ubiquitination is inhibited by DUBs, which either directly remove ubiquitins from substrates (B) or block the ubiquitination process. (D) DUBs cleave ubiquitin precursor proteins to release free ubiquitin molecules, which are further used for ubiquitination.

Accumulative studies have shown that the pathological processes of CVDs are closely regulated by ubiquitination, a post‐translational modification (PTM) that is widely involved in signal transduction, inflammatory responses, metabolism, and cell fate determination.^11^, ^12^, ^13^, ^14^, ^15^, ^16^, ^17^ In the process of ubiquitination, ubiquitin, a 76‐amino acid small protein, is covalently conjugated to a lysine residue of the substrate protein under the sequential catalysis of ubiquitin‐activating enzymes (E1s), ubiquitin‐conjugating enzymes (E2s), and ubiquitin ligases (E3s)^18^ (Figure 1A). First, an E1 hydrolyzes ATP and forms a thioester bond between the sulfhydryl group of its active cysteine and the carboxyl group of the ubiquitin C‐terminal glycine. Second, the activated ubiquitin is attached to an active cysteine of the E2 through a new E2‐ubiquitin thioester bond. Then, the E2‐ubiquitin complex interacts with an E3, which recognizes the protein substrate to mediate the final ubiquitin transfer.^18^ E3s containing the really interesting new gene (RING) domain catalyze the direct transfer of ubiquitin from the E2 to the substrate. In contrast, E3s belonging to the RING‐between‐RING (RBR) and homologous to the E6AP carboxyl terminus (HECT) subfamilies form a ubiquitin‐E3 thioester intermediate before transferring the ubiquitin to the substrate^18^, ^19^ (Figure 1A). According to the type of ubiquitin attachment, ubiquitination can be classified as polyubiquitination, monoubiquitination, and multi‐monoubiquitination (Figure 1A). In polyubiquitin chains, ubiquitin monomers are linked together via isopeptide bonds between the C‐terminal glycine carboxyl group of one ubiquitin and the amino group of the N‐terminal methionine residue (M1) or any one of the internal lysine residues (K6, K11, K27, K29, K33, K48, K63) in the next ubiquitin.^20^ Of note, polyubiquitin chains can be added to substrates by “sequential addition” or “en bloc transfer.” In the sequential addition, individual ubiquitin monomers are transferred stepwise to the end of a growing polyubiquitin chain. By contrast, in the en bloc mechanism, a pre‐assembled polyubiquitin chain is transferred as a whole to a substrate.^21^ Functionally, ubiquitination controls the localization, activity, stability, or binding partners of protein substrates. As a reversible PTM, ubiquitination is antagonized by deubiquitinating enzymes (DUBs), which are divided into seven subfamilies based on sequence and domain conservation: ubiquitin‐specific proteases (USPs), ovarian tumor proteases (OTUs), ubiquitin C‑terminal hydrolases (UCHs), Machado–Josephin domain‐containing proteases (MJDs), JAB1/MPN/MOV34 family (JAMMs), motif interacting with ubiquitin‐containing novel DUB family (MINDYs), and zinc finger with UFM1‐specific peptidase domain protein (ZUFSP/ZUP1).^22^, ^23^ Mostly, DUBs directly remove ubiquitin molecules from protein substrates (Figure 1B). However, OTUB1, a DUB of the OTU subfamily, possesses a non‐canonical function that enables it to obstruct the ubiquitination of protein targets by blocking ubiquitin transfer from E2s^24^, ^25^, ^26^ (Figure 1C). Of note, the removed ubiquitin molecules from substrates will be recycled for new ubiquitination processes. Besides, DUBs can also enrich the free ubiquitin pool by cleaving ubiquitin precursors encoded by *UBA52*, *RPS27A*, *UBB*, and *UBC* to release free ubiquitin molecules^27^ (Figure 1D).

Ubiquitin‐modifying enzymes (UMEs) comprising E1s, E2s, E3s, and DUBs orchestrate the diverse and exquisite ubiquitination of target proteins to precisely control their cellular localization, protein interaction, enzymatic activity, and degradation, thereby affecting the initiation and/or outcome of brain diseases. For example, loss‐of‐function mutations in the *TRIM37* gene, which encodes the E3 ligase TRIM37, cause the Mulibrey (muscle–liver–brain–eye) nanism.^28^, ^29^ In the scope of CVDs, the gene encoding the E3 ligase RNF213 has been identified as the most prominent susceptibility gene for MMD.^30^, ^31^, ^32^, ^33^, ^34^ In this review, we elaborate on the functional roles and molecular mechanisms of UMEs in CVDs and discuss the possibility of exploring UMEs as therapeutic targets for these diseases.

### 1.1 UMEs regulate cerebral injury after ischemic stroke

Stroke is the second leading cause of death and disability worldwide. Around the world, there are more than 12 million new stroke cases per year and approximately 100 million people are living with the aftermath of a stroke.^10^ Stroke is caused by infarction or rupture of a blood vessel in the brain, and accordingly can be classified into ischemic stroke and hemorrhagic stroke, with the former accounting for nearly 80% of all stroke cases.^35^, ^36^ Thrombolytic therapy with tissue plasminogen activator (tPA) and mechanical thrombectomy are two common ways to restore blood supply after ischemic stroke.^37^ However, blood reperfusion can cause secondary brain damage known as cerebral ischemia/reperfusion (CI/R) injury. Multiple mechanisms, such as calcium overload, excitotoxicity, mitochondrial damage, oxidative stress, blood‐brain barrier (BBB) disruption, and inflammation, are involved in CI/R injury, culminating in neuronal cell death and neurological deficits.^38^ The middle cerebral artery occlusion (MCAO) model, an animal model phenocopying key features of human ischemic stroke, is widely used to study the pathophysiology and treatment of ischemic stroke. With the MCAO model, regulatory functions of UMEs in ischemic stroke injury have been revealed. Recent studies have shown that UMEs affect the injury in ischemic stroke predominantly by regulating neuronal death, axonal function, neuroinflammation, BBB integrity, and mitochondrial dysfunction.

#### 1.1.1 UMEs regulate neuronal death after ischemic stroke

**FIGURE 2:**
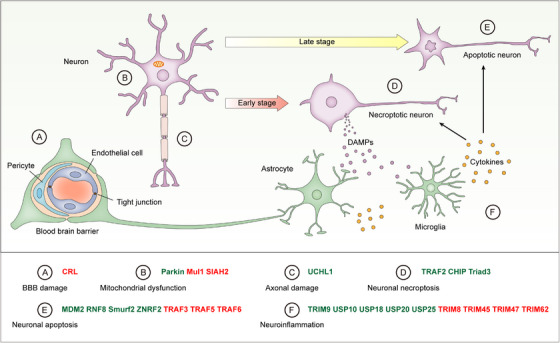
The role of UMEs in ischemic stroke. Ischemic stroke induces BBB damage, mitochondrial dysfunction, axonal damage, and neuronal death. Shortly after cerebral ischemia/reperfusion, neurons undergo necroptosis, leading to the release of DAMPs including S100A8/A9 and HMGB1. These DAMPs stimulate adjacent astrocytes and microglia to produce pro‐inflammatory cytokines. Neuroinflammation in turn promotes neuronal necroptosis in a positive feedback loop and instigates neuronal apoptosis in the late stage. UMEs influence ischemic stroke injury by regulating BBB damage (A), mitochondrial dysfunction (B), axonal damage (C), neuronal necroptosis (D), neuronal apoptosis (E), and neuroinflammation (F). UMEs inhibiting ischemic stroke injury are in green and UMEs promoting ischemic stroke injury are in red.

Neuronal cell death is the basic pathophysiology of stroke, and the ischemic insult sequentially induces two major forms of programmed cell death, that is, necroptosis and apoptosis^39^ (Figure 2). Necroptosis is a regulated necrosis that is rapidly induced in neurons shortly after cerebral ischemia.^39^ In response to necroptosis‐inducing stimuli, receptor‐interacting protein kinase 1 (RIPK1) undergoes a conformational change and recruits RIPK3 to form a RIPK1/RIPK3 oligomer. In this kinase complex, RIPK3 is activated and then phosphorylates the effector molecule mixed lineage kinase domain‐like protein (MLKL).^40^ Phosphorylated MLKL forms oligomers that translocate to the plasma membrane. At the membrane, MLKL undergoes conformational changes, leading to rapid breakage of the cell membrane and cell death.^41^, ^42^ Due to membrane permeabilization, necroptotic cells leak intracellular contents comprising damage‐associated molecular patterns (DAMPs), which further activate innate immune responses to incite inflammation^43^ (Figure 2). Inhibition of necroptosis has been shown to be neuroprotective in mice subjected to MCAO.^44^, ^45^ Necroptosis is inhibited by E3 ligases TRAF2, CHIP, and Triad3, and they can mitigate cerebral ischemic injury by attenuating necroptosis and neuroinflammation.^46^, ^47^, ^48^ Consistently, viral vector‐mediated overexpression of CHIP has been shown to prevent neuronal cell death after cerebral ischemia.^49^

In mice, MCAO‐induced neuronal cell death undergoes the transition from necroptosis to apoptosis over time, and apoptosis becomes the main type of neuronal cell death following the initial necroptosis^39^ (Figure 2). Ischemia‐induced neuronal apoptosis is inhibited by UMEs such as MDM2, RNF8, Smurf2 and ZNRF2, and enhanced by TRAF3, TRAF5, and TRAF6.^11^, ^50^, ^51^, ^52^, ^53^, ^54^, ^55^ Cerebral ischemia induces the upregulation of MDM2, an E3 ligase that negatively regulates p53 via both repressing p53 target gene transcription and ubiquitinating p53 for degradation.^50^ Consistent with studies showing that p53 promotes stroke‐induced apoptosis and affects functional recovery after stroke,^56^, ^57^ the single‐nucleotide polymorphism of the *MDM2* gene (SNP309T > G), which enhances MDM2 expression, is associated with better functional outcomes in patients with ischemic or hemorrhagic stroke.^50^ RNF8 is an E3 ligase that is involved in DNA damage repair via histone ubiquitination, and ablation of RNF8 leads to DNA damage accumulation and neuronal apoptosis.^58^, ^59^ In mice subjected to MCAO, RNF8 plays a neuroprotective role by inducing the ubiquitination and degradation of HDAC2, which enhances oxygen‐glucose deprivation (OGD)‐induced neuronal apoptosis via regulating GSK3β activation.^51^ Smurf2 is another E3 ligase that can inhibit neuronal apoptosis induced by cerebral ischemia and OGD, and overexpression of Smurf2 reduces brain injury in mice subjected to MCAO.^11^ Smurf2 ubiquitinates Yin Yang 1 (YY1) for proteasome‐dependent degradation, thereby suppressing apoptosis via inactivating the apoptosis‐inducing YY1/HIF1α/DDIT4 axis.^11^ CI/R‐induced neuronal apoptosis can also be inhibited by the E3 ligase ZNRF2, which inhibits apoptosis by preventing excessive autophagy, and overexpression of ZNRF2 attenuates cerebral injury in rats after MCAO.^52^ In sharp contrast to the aforementioned apoptosis‐inhibiting E3 ligases, several E3 ligases of the tumor necrosis factor receptor‐associated factor (TRAF) family, including TRAF3/5/6 can enhance CI/R‐induced neuronal apoptosis.^53^, ^54^, ^55^ For example, TRAF6 potentiates CI/R‐induced neuronal apoptosis by K63 ubiquitinating and activating Rac1.^54^

#### 1.1.2 UME regulates axonal function after ischemic stroke

In addition to grey matter, white matter can also be injured by ischemic stroke.^60^, ^61^ UCHL1 is a neuron‐specific DUB that is essential for axonal function.^14^ After cerebral ischemia, UCHL1 is deactivated by reactive lipids, which bind to the C152 residue of UCHL1, leading to an impaired ubiquitin‐proteasome pathway. However, the UCHL1 C152A mutant preserves the ubiquitin hydrolase activity in the presence of reactive lipids.^62^ As compared with wild‐type controls, the UCHL1 C152A knock‐in mice show decreased accumulation of ubiquitinated proteins and axonal injury after MCAO, suggesting that UCHL1 plays a critical role in maintaining axonal function after ischemic stroke.^14^

#### 1.1.3 UMEs regulate neuroinflammation after ischemic stroke

**FIGURE 3:**
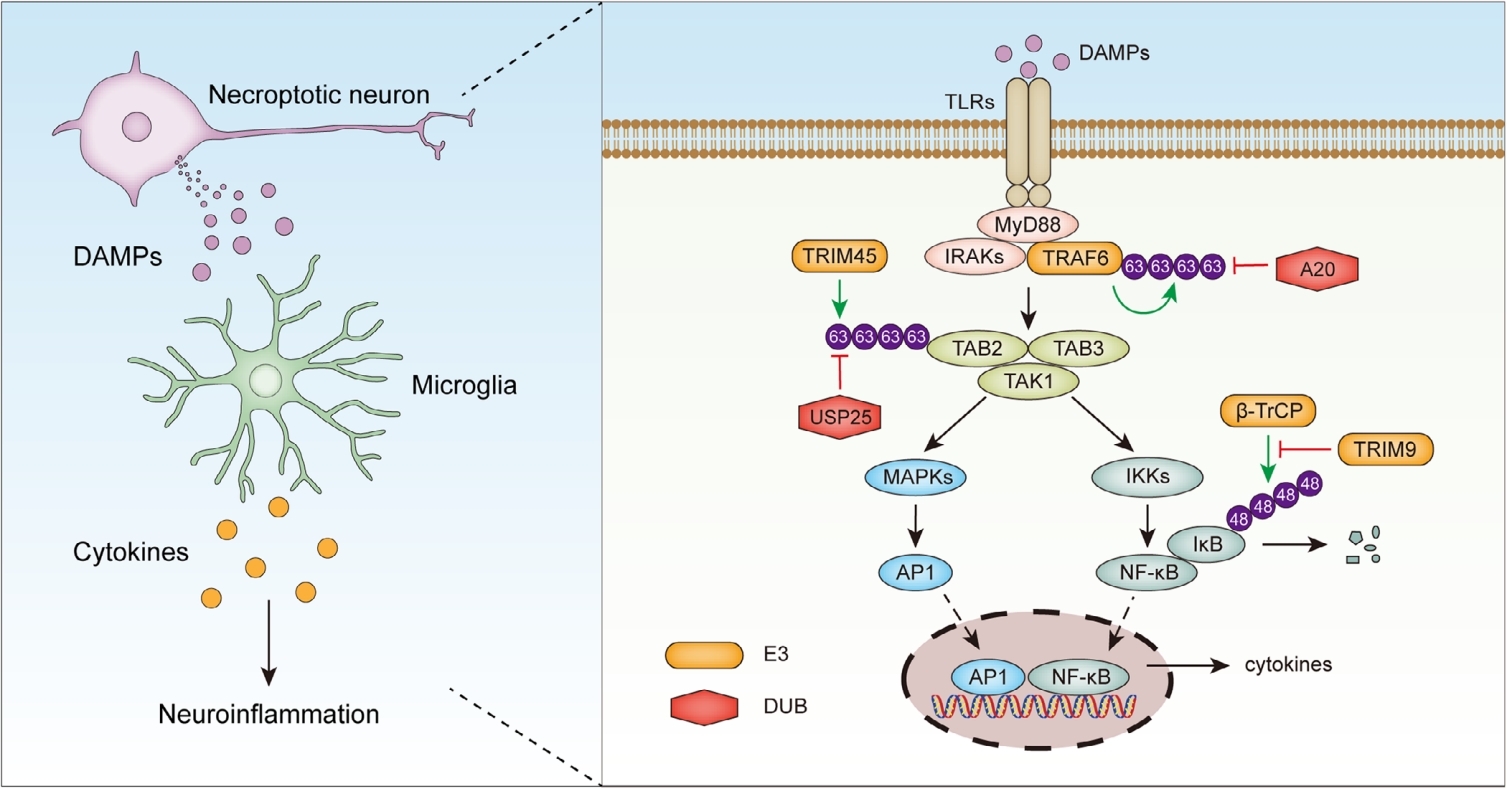
UMEs regulate neuroinflammatory signal transduction after ischemic stroke. DAMPs released from necroptotic neurons induce the production of pro‐inflammatory cytokines in glial cells mainly by activating NF‐κB and MAPK signaling pathways. E3s and DUBs tightly control the activity of these signaling pathways, thereby affecting neuroinflammation and ischemic stroke outcomes.

Neuroinflammation is an indispensable component of the pathological machinery in ischemic stroke.^63^ Shortly after ischemic stroke, DAMPs such as S100A8/A9 and HMGB1 are released from necroptotic cells. These DAMPs are recognized by microglia and astrocytes, two innate immune cell populations in the brain, through pattern recognition receptors, resulting in the production of pro‐inflammatory cytokines and chemokines^39^, ^64^ (Figure 2). The post‐stroke neuroinflammation is driven by various pro‐inflammatory signaling pathways, in particular the nuclear factor‐kappa B (NF‐κB) pathway, which is tightly regulated by ubiquitination and UMEs.^63^, ^65^, ^66^, ^67^, ^68^ Noteworthy, in response to pro‐inflammatory stimuli, polyubiquitin chains catalyzed by UMEs provide large scaffolds to induce multi‐protein structures comprising IκB kinases (IKKs), which serve as an upstream organizing center regulating NF‐κB activation.^69^, ^70^ As such, multiple UMEs have been shown to influence ischemic stroke injury by regulating neuroinflammation (Figure 3). Ischemic stroke‐induced neuroinflammation has been shown to be promoted by TRIM8,^71^ TRIM45,^72^ TRIM47^73^ and TRIM62,^74^ and inhibited by TRIM9,^13^ USP10,^75^ USP18,^76^ USP20,^77^ and USP25.^78^ For example, after CI/R injury, microglia‐mediated neuroinflammation and neurological deficit are enhanced by the E3 ligase TRIM45.^72^ After OGD/R, TRIM45 catalyzes K63‐specific polyubiquitination on TAB2, which is crucial for the phosphorylation of TAK1 and the subsequent activation of NF‐κB signaling. Moreover, microglia‐specific knockdown of TRIM45 significantly mitigates neurological deficit following CI/R injury in mice.^72^ In sharp contrast to TRIM45, the DUB USP25 inhibits CI/R‐induced K63 ubiquitination of TAB2 in microglia.^78^ In both mice and humans, microglial expression of USP25 is upregulated in the ischemic penumbra.^78^ USP25 physically interacts with TAB2 through the UIM2 domain and cleaves K63 polyubiquitin chains on TAB2. In mice, ablation of USP25 significantly exacerbated MCAO‐induced cerebral deficits by enhancing neuroinflammation.^78^ Ubiquitination and degradation of IκBα, the inhibitor that retains NF‐κB in the cytoplasm in resting cells, is essential for the activation of NF‐κB signaling. Of note, the ubiquitination of IκBα is induced by an E3 ligase complex comprising β‐TrCP.^79^ As a counter‐regulating mechanism, the degradation of IκBα is inhibited by TRIM9, which competes with IκBα for β‐TrCP interaction and thereby inhibits the ubiquitination of IκBα.^80^ Upon ischemic stroke, TRIM9 inhibits NF‐κB‐mediated neuroinflammation by stabilizing IκBα, resulting in alleviated cerebral damage.^13^

#### 1.1.4 UMEs regulate BBB integrity after ischemic stroke

BBB disruption, characterized by loss of BBB junctional proteins and enhanced permeability, is another pathological process associated with cerebral ischemic stroke. Disrupted BBB subsequently leads to cerebral edema and neuronal cell death.^81^ After ischemic stroke, BBB damage is promoted by the E3 ubiquitin ligase CRL, which induces the degradation of the protective protein neurofibromatosis 1 (NF1).^82^ The activity of CRL is inhibited by the small‐molecular inhibitor MLN4924, and treatment of mice with MLN4924 ameliorates ischemic brain injury by inducing the accumulation of NF1.^82^ In addition to neuronal apoptosis, BBB disruption is also mitigated in TRAF5 knockout mice after CI/R, indicating a role of TRAF5 in regulating BBB damage.^53^

#### 1.1.5 UMEs regulate mitochondrial dysfunction after ischemic stroke

Mitochondrial dysfunction is a key mechanism contributing to brain injury in ischemic stroke.^83^ Mul1 is a mitochondrial membrane protein with dual E3 ligase functions in both ubiquitination and sumoylation, a ubiquitination‐like PTM.^84^, ^85^ Mul1 is upregulated in the rat brain after MCAO, and it aggravates mitochondrial dysfunction by regulating the protein abundance of the mitochondrial fission protein Drp1 and the mitochondrial fusion protein Mfn2 through sumoylation and ubiquitination, respectively.^84^ Knockdown of Mul1 ameliorates MCAO‐induced brain injury by restoring protein abundance of Drp1 and Mfn2.^84^ In response to OGD, the E3 ligase SIAH2 is activated in neurons, and it induces the ubiquitination and degradation of mitochondrial NCX3, a protein essential for mitochondrial integrity and neuronal survival during hypoxia. As compared with control neurons, SIAH2‐deficient neurons show improved mitochondrial function under OGD conditions due to elevated NCX3 levels.^86^ Another study demonstrated that SIAH2 could also aggravate ischemia‐induced mitochondrial damage by inducing the ubiquitination and proteasomal degradation of AKAP121, a mitochondrial scaffold protein essential for mitochondria activity.^87^ Therefore, the two studies jointly show that SIAH2 contributes to mitochondrial damage upon ischemic stress.^86^, ^87^ Selective mitochondrial autophagy, known as mitophagy, serves as a key mechanism in clearing damaged mitochondria and it is activated in ischemic brains.^88^ Upon ischemic injury, the E3 ligase Parkin is recruited to the damaged mitochondria and ubiquitinates mitochondrial membrane proteins to trigger mitophagy.^89^ In mice, Parkin‐mediated mitophagy has been shown to be a key protective mechanism in CI/R injury.^88^, ^90^

In aggregate, these studies show that, after ischemic stroke, UMEs impinge on cerebral injury by regulating a broad range of biological activities, implying that UMEs may serve as potential therapeutic targets for ischemic stroke.

### 1.2 UMEs regulate cerebral injury after hemorrhagic stroke

Nearly 20% of stroke cases are hemorrhagic, with intracerebral hemorrhage (ICH) accounting for about 10−15%.^91^, ^92^ ICH causes neuronal cell death and neurological deficits by hematoma‐associated mechanical damage and secondary injury mechanisms such as oxidative stress, mitochondrial dysfunction, neuronal excitotoxicity, calcium overload, neuroinflammation, and free radical production.^93^ UMEs have been shown to affect the outcome of ICH by regulating many of these pathological processes.^94^, ^95^, ^96^, ^97^, ^98^ The DUB A20, encoded by the *TNFAIP3* gene, serves as a brake on inflammatory responses via suppressing multiple pro‐inflammatory signaling pathways, such as NF‐κB and JAK‐STAT signaling^66^, ^99^ (Figure 3). Mutations in or close to the *TNFAIP3* gene are associated with various autoimmune diseases including systemic lupus erythematosus, rheumatoid arthritis, multiple sclerosis, and colitis.^66^, ^100^, ^101^ ICH‐induced inflammatory injury is also inhibited by A20, and overexpression of A20 ameliorates brain damage after ICH.^94^ Moreover, in humans, A20 mRNA levels in peripheral blood mononuclear cells are negatively correlated with neurological deficits after ICH, indicating that A20 is a key suppressor for ICH injury.^94^ Mitochondrial dysfunction and oxidative stress are inhibited by PGC‐1α and enhanced by RNF34, an E3 ligase inducing the ubiquitination‐mediated degradation of PGC‐1α.^95^, ^102^ Overexpression of RNF34 exacerbates ICH‐induced brain injury by promoting PGC‐1α protein degradation and increasing oxidative stress and mitochondrial dysfunction.^95^ Necroptosis is an important mechanism causing brain injury after ICH and it can be regulated by CHIP, which ubiquitinates the key component of necroptosis, RIPK3, for lysosomal degradation.^103^, ^104^, ^105^ Overexpression of CHIP inhibits neuronal necroptosis and neuroinflammation in rats after ICH, resulting in reduced hemorrhagic lesions. Concordantly, CHIP deficiency leads to aggravated brain injury after ICH.^96^ In addition to necroptosis, apoptosis is also induced by ICH, and it can be enhanced by the DUBs USP4 and USP11.^97^, ^98^

Subarachnoid hemorrhage (SAH) is another subtype of hemorrhagic stroke, accounting for nearly 5−10% of acute stroke.^92^, ^106^ The E3 ligase RNF216, also known as Triad3A, has been shown to modulate synaptic plasticity in glutamatergic neurons by inducing the ubiquitination and degradation of Arc.^107^ Upon SAH, RNF216 increases oxyhemoglobin‐induced intracellular Ca^2+^ accumulation in neurons by restraining the Arc‐AMPAR pathway, thereby promoting cytotoxicity and neuronal apoptosis. Moreover, the downregulation of RNF216 ameliorates brain injury following SAH.^108^ In addition to RNF216, neuronal apoptosis induced by SAH is also enhanced by the E3 ligase TRAF3, which enhances SAH‐induced NF‐κB and MAPK signaling by activating TAK1.^109^ Inflammation is a key pathological process contributing to early brain injury (EBI) following SAH. After experimental SAH, microglia upregulate the expression of Peli1, an E3 ligase that positively regulates neuroinflammation by promoting c‐IAP2 ubiquitination and downstream inflammatory signaling in microglia.^65^, ^110^ Consistently, the knockdown of Peli1 reduces neuroinflammation and improves neurological outcomes during EBI after SAH.^110^

### 1.3 UMEs regulate moyamoya disease (MMD)

MMD is an idiopathic cerebral vasculopathy characterized by progressive narrowing of the intracranial portion of the internal carotid artery and its main branches including the middle cerebral and anterior cerebral arteries.^7^, ^111^ In MMD, a hazy network of collateral arteries named moyamoya vessels develops around the occlusive region to compensate for the blood flow. MMD usually causes cerebral ischemia in pediatric and adult patients, but half of adult patients can also develop intracranial bleeding.^112^ Therefore, MMD poses a key risk factor for both hemorrhagic and ischemic stroke.^111^, ^112^

The annual incidence of MMD is as high as 0.5–1.5/100 000 in East Asian countries including China, Japan, and Korea, but as low as 0.1/100 000 in other parts of the world.^7^ The difference in MMD prevalence is largely due to genetic susceptibility factors in East Asian populations. Indeed, *RNF213*, which encodes the E3 ligase RNF213, was identified as the principal susceptibility gene for MMD.^7^, ^30^, ^31^ The heterozygous p.Arg4810Lys variant of *RNF213* has been identified as a founder mutant present in East Asian MMD patients.^30^, ^32^, ^33^, ^34^ Around 1.5% of the population of South Korea and Japan carry this variant, but it is rarely found in Caucasians, which may explain, at least partly, the nearly tenfold higher frequency of MMD in East Asian countries than in other regions.

RNF213 is the biggest E3 ligase in the human proteome with a mass of 591 kD, consisting of an N‐terminal stalk, a dynein‐like ATPase core, and a C‐terminal multidomain E3 module.^113^ Since most of the pathological MMD variants map to the E3 module of RNF213, these MMD‐related variants may disturb the E3 ligase activity of RNF213.^113^ Consistently, a recent study found that proteins encoded by MMD‐associated *RNF213* variants, including the most prevalent Arg4810Lys variant, had reduced ubiquitination activity, suggesting that decreased E3 ligase activity of RNF213 contributes to the pathogenesis of MMD.^114^ A recent study found that ablation of RNF213 disrupted the barrier function of human cerebral endothelium in vitro, which could be a potential pathogenic mechanism causing MMD.^15^ However, the exact biological functions and molecular mechanisms of RNF213 in MMD remain largely unclear. In the future, studies on macromolecular interactions, conformational dynamics, and biochemical functions of RNF213 may reveal the role and mechanism of action of this E3 in MMD and accelerate the development of RNF213‐targeting therapies for MMD.

### 1.4 UMEs regulate atherosclerosis

Atherosclerosis is a chronic vascular disease resulting from the complex interplay between lipid metabolism and immune responses. Besides, atherosclerosis can also contribute to the development of other diseases of the circulation system, such as coronary artery disease, peripheral artery disease, and stroke.^6^, ^115^, ^116^ The chronic build‐up of atherosclerotic plaques in the sub‐endothelial intimal layer of medium‐ and large‐sized arteries causes stenosis and restricts blood flow to critical organs, particularly the brain. In addition, rupture of the atherosclerotic plaque leads to acute thrombo‐occlusive events, including ischemic stroke.^117^ The p.Arg4810Lys variant of *RNF213*, which represents the most prevalent genetic abnormality in East Asian MMD patients, is also closely associated with intracranial atherosclerosis and ischemic stroke.^118^, ^119^, ^120^, ^121^ Besides, this *RNF213* variant predisposes patients with symptomatic intracranial atherosclerosis to stroke recurrence.^122^

**FIGURE 4:**
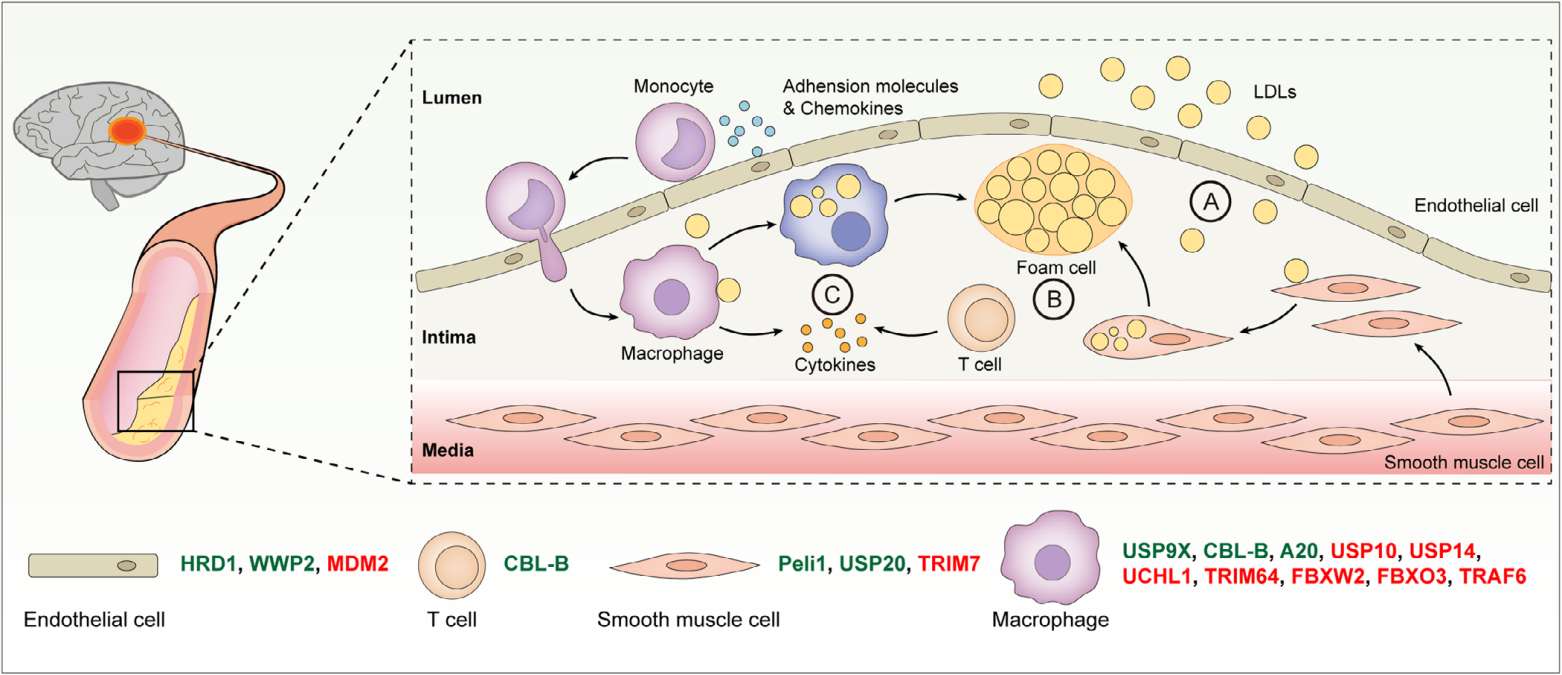
The role of UMEs in atherosclerosis. Atherosclerosis is a primary risk factor for stroke. During the initial stages of atherosclerosis, LDLs are transported across dysfunctional endothelial cells to the sub‐endothelial space of arteries (A). LDLs in the artery intima are engulfed by macrophages through scavenger receptors. After ingesting overdose LDLs, macrophages laden with lipids become foam cells. Apart from macrophages, smooth muscle cells can also become foam cells after ingesting LDLs (B). Accumulation of foam cells further leads to the formation of atherosclerotic plaques. Besides, plaque formation is strongly promoted by pro‐inflammatory cytokines produced by macrophages and T cells (C). UMEs can influence the pathogenesis and development of atherosclerosis by regulating various cell populations including endothelial cells, T cells, macrophages, and smooth muscle cells. UMEs inhibiting atherosclerosis are in green and UMEs promoting atherosclerosis are in red.

**FIGURE 5:**
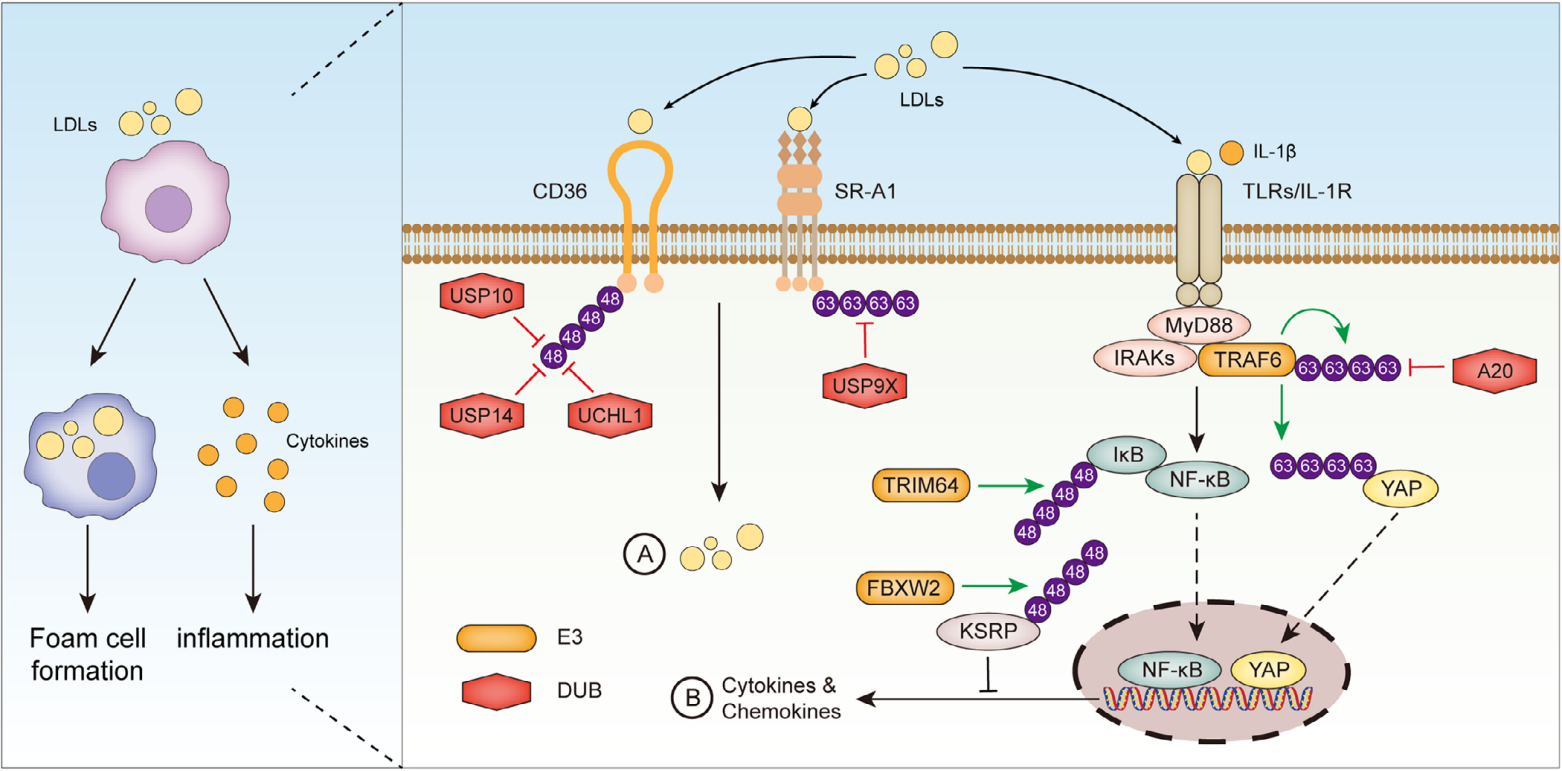
UMEs regulate macrophage functions in atherosclerosis. Macrophages are a key cell population promoting atherosclerosis. On the one hand, macrophages ingest LDLs to become foam cells (A). On the other hand, macrophages produce pro‐inflammatory cytokines in response to LDLs (B). The two processes are closely controlled by UMEs.

Atherosclerosis is a cholesterol‐related disease caused by the deposition of lipoproteins, especially low‐density lipoproteins (LDLs), in the intimal space of arteries (Figure 4). In the intima, LDLs are oxidized by free radicals to form oxidized LDLs (OxLDLs), which can be taken up primarily by macrophages through scavenger receptors (SRs).^123^, ^124^ Macrophages and vascular smooth muscle cells (VSMCs) engulfing excessive OxLDLs differentiate into foam cells, and the accumulation of foam cells contributes to the development of atherosclerotic fatty streaks and plaques.^125^, ^126^ In addition to driving the transition of macrophages and VSMCs to foam cells, OxLDLs as a group of metabolism‐associated molecular patterns (MAMPs) can also promote atherosclerosis by triggering inflammation, which is a key pathological process underlying the pathogenesis and progression of atherosclerosis^123^ (Figures 4 and 5). UMEs have emerged as key regulators of atherosclerosis and they affect the onset and progression of atherosclerosis by modulating endothelial cell function, foam cell formation, and vascular inflammation.

#### 1.4.1 UMEs regulate endothelial cell function in atherosclerosis

The atherosclerotic process begins with the accumulation of LDLs in the sub‐endothelial space of arteries^127^ (Figure 4A). Endothelial cell dysfunction, such as altered permeability and apoptosis, is the critical initial step in atherogenesis, and this process is tightly regulated by E3 ligases HRD1, MDM2, and WWP2.^128^, ^129^, ^130^ The binding of OxLDLs with lectin‐like oxidized LDL receptor‐1 (LOX‐1), the specific scavenger receptor for OxLDLs on endothelial cells, induces endothelial dysfunction and OxLDL uptake.^131^ LOX‐1 can be ubiquitinated by the E3 ligase HRD1 for degradation.^128^ HRD1 expression is downregulated in human atherosclerotic intima and its overexpression attenuates OxLDL‐induced apoptosis of endothelial cells by reducing LOX‐1 abundance, indicating that decreased HRD1 expression induces endothelial dysfunction in atherosclerosis.^128^ Oxidative stress is a primary driving factor in endothelial dysfunction. The E3 ligase MDM2 promotes OxLDL‐induced mitochondrial damage and oxidative stress in endothelial cells.^129^ MDM2 induces the UPS‐dependent degradation of retinoid X receptor beta (RXRβ), a protein that plays a protective role in endothelial cells upon OxLDL stimulation. In LDLr^−/−^ mice, pharmacological inhibition of MDM2 increases the protein abundance of RXRβ in the aorta and decreases the formation of atherosclerotic lesions.^129^ In sharp contrast, the E3 ligase WWP2 can inhibit OxLDL‐induced endothelial cell injury by antagonizing oxidative stress. Mechanistically, WWP2 ubiquitinates PDCD4 for degradation, thereby activating the antioxidant HO‐1 pathway in endothelial cells. In ApoE^−/−^ mice, overexpression of WWP2 ameliorates atherosclerosis by reducing oxidative stress and inflammation.^130^

#### 1.4.2 UMEs regulate foam cell formation in atherosclerosis

Foam cell formation is a hallmark of atherosclerosis (Figure 4B). A majority of foam cells are derived from macrophages, which ingest OxLDLs through scavenger receptors SR‐A1 and SR‐B2 (CD36)^124^, ^132^ (Figure 5A). Upon binding with OxLDLs, SR‐A1 is K63 polyubiquitinated at the K27 residue, and this PTM facilitates SR‐A1 internalization, OxLDL uptake, and foam cell formation.^133^ The K63‐linked polyubiquitination of SR‐A1 is counter‐regulated by the DUB USP9X (Figure 5A). Pharmacological or genetic inhibition of USP9X increases OxLDL‐induced SR‐A1 ubiquitination and internalization in macrophages. Furthermore, disrupting the interaction between SR‐A1 and USP9X with a cell‐penetrating peptide exacerbates atherosclerosis by increasing foam cell formation, showing that USP9X is an important beneficial regulator of atherosclerosis.^133^ The other key scavenger receptor CD36 can also be ubiquitinated, and the ubiquitination of CD36 leads to its proteasomal degradation.^134^, ^135^ Of note, the ubiquitination and degradation of CD36 is inhibited by DUBs including USP10, USP14, and UCHL1^136^, ^137^, ^138^ (Figure 5A). Inhibition of USP10, USP14 or UCHL1 reduces CD36 protein abundance in macrophages and thereby diminishes OxLDL‐induced foam cell formation.^136^, ^137^, ^138^ Apart from macrophages, another important source of foam cells is VSMCs. The E3 ligase TRIM7 promotes the proliferation and migration of VSMCs in atherosclerosis, and the downregulation of TRIM7 alleviates atherosclerosis in ApoE^−/−^ mice.^139^ On the contrary, the E3 ligase Peli1 inhibits atherosclerosis progression by reducing inflammation and the transition of VSMCs to foam cells.^140^

#### 1.4.3 UMEs regulate inflammation in atherosclerosis

Atherosclerosis is characterized by continuous low‐grade inflammation in the artery wall, and inflammation accelerates plaque expansion and destabilization^141^, ^142^ (Figure 4C). Macrophages are the predominant source of pro‐inflammatory molecules in atherosclerosis and macrophage‐mediated inflammatory responses are regulated by UMEs including A20, TRIM64, FBXW2, FBXO3, TRAF6^143^, ^144^, ^145^, ^146^, ^147^ (Figure 5B). A20, a special UME with both DUB and E3 ligase activities, is an NF‐κB inhibitor and critically regulates inflammatory responses in various diseases.^99^, ^148^, ^149^ A20 was found to play a protective role in atherosclerosis by suppressing the expression of NF‐κB target genes including cytokines and adhesion molecules.^143^ As compared with control ApoE^−/−^ mice, atherosclerotic lesions are increased in A20‐haploinsufficient mice and decreased in A20‐overexpressing mice.^143^ In contrast, OxLDL‐induced NF‐κB‐dependent inflammation in macrophages is promoted by the E3 ligase TRIM64, which enhances IκBα degradation by ubiquitinating IκBα at the K67 residue.^144^ The E3 ligase FBXW2 is an F‐box protein and acts as a substrate‐binding component of the E3 ligase complex termed Skp1‐Cullin‐F‐box protein (SCF) complex.^145^ FBXW2 is upregulated in macrophages in atherosclerotic plaques. FBXW2 enhances the production of pro‐inflammatory factors by mediating the ubiquitination and degradation of KSRP, an RNA‐binding protein that negatively regulates the synthesis of a subset of cytokines and chemokines. Consistently, myeloid cell‐specific ablation of FBXW2 mitigates atherosclerosis in mice, accompanied by reduced expression of pro‐inflammatory factors in atherosclerotic lesions.^145^ Another F‐box protein of the SCF complex, FBXO3, can also promote atherosclerosis by enhancing inflammation.^146^ FBXO3 is predominantly expressed in macrophages in human carotid atherosclerotic plaques, and FBXO3 depletion in macrophages diminishes OxLDL‐induced inflammatory responses. Intriguingly, individuals carrying a hypo‐functioning FBXO3 variant are less susceptible to atherosclerosis.^146^ YAP is an essential signaling molecule of the Hippo pathway and it was recently shown to exacerbate atherosclerosis by promoting chemokine production in macrophages.^147^ YAP expression is upregulated in macrophages in mouse and human atherosclerotic lesions, and myeloid cell‐specific YAP overexpression aggravates atherosclerosis in mice. Upon stimulation with IL‐1β, a key pro‐inflammatory cytokine involved in atherosclerosis, YAP is K63 ubiquitinated by the E3 ligase TRAF6 at the K252 residue, leading to its protein stabilization and nuclear translocation. This study found that IL‐1β enhanced YAP‐mediated chemokine production in macrophages by activating TRAF6, highlighting a pivotal role of TRAF6 in the inflammation‐driven progression of atherosclerosis.^147^

Akin to macrophages, T cells are a dominant immune cell type in atherosclerotic plaques and mediate the inflammatory responses underlying atherosclerosis^150^, ^151^ (Figure 4C). In both human and mouse atherosclerotic plaques, the E3 ligase CBL‐B is mainly expressed in infiltrating macrophages and T cells.^152^ CBL‐B deficiency exacerbates vascular inflammation in mice by increasing the abundance and cytotoxicity of CD8^+^ T cells as well as macrophage activation, resulting in aggravated atherosclerosis.^152^ In addition to macrophages and T cells, VSMCs can also contribute to inflammation in atherosclerosis. The DUB USP20 has been shown to inhibit IL‐1‐ and TNF‐evoked inflammatory responses in VSMCs by deubiquitinating RIPK1. In vivo, specific overexpression of USP20 in VSMCs significantly reduces vascular inflammation and ameliorates atherosclerosis.^153^

Collectively, these reports demonstrate that UMEs regulate various key aspects in the pathogenesis and progression of atherosclerosis. Therefore, enhancing the beneficial functions and/or inhibiting the detrimental functions of UMEs may impede the progression of atherosclerosis, preventing the occurrence of more severe CVDs such as stroke.

### 1.5 UMEs as therapeutic targets and tools

Considering that UMEs serve as versatile and critical regulators in CVDs, potent and specific UME inhibitors/agonists may become efficacious drugs for the prevention and treatment of CVDs. For example, the USP14 inhibitor IU1 has been shown to attenuate neurological deficits caused by ischemic stroke.^154^, ^155^ Since inhibition of USP14 diminishes foam cell formation, IU1 may also ameliorate atherosclerosis.^137^ Of note, compared with E1, E2, and E3 ubiquitinating enzymes, DUBs are more favorable targets for the development of small‐molecule inhibitors.^156^, ^157^ In the past two decades, DUBs have emerged as novel drug targets for cancer and immune disorders.^158^ In the foreseen future, UME inhibitors, particularly DUB inhibitors, may also enrich the therapeutic armamentarium for CVDs. Notably, in the NF‐κB signaling, which critically regulates inflammation and cell death in multiple CVDs, stimulus‐specific polyubiquitin scaffolds provide the docking sites for key upstream signaling molecules including IKKs.^69^, ^70^ In light of this, compared with the conventional “target‐centric” inhibitors that inhibit single UMEs, “network‐centric” inhibitors, which inhibit ubiquitin‐mediated assembly of signaling complexes, may be more specific and effective.^159^ In addition, given that UMEs tightly control the abundance, location, and activation of key proteins involved in CVDs, UMEs can also be applied to treat CVDs by precisely inhibiting detrimental proteins and enhancing beneficial proteins. Indeed, techniques based on UMEs, such as deubiquitinase‐targeting chimera (DUBTAC) and proteolysis‐targeting chimera (PROTAC), are gaining increasing attention as innovative therapeutic methods.^160^, ^161^, ^162^ Therefore, UMEs may become novel drug targets and therapeutic tools, opening up new possibilities for the prevention and treatment of CVDs.

## 2 CONCLUSION AND PERSPECTIVE

CVDs are a leading cause of disability and death in both developing and developed countries. In 2020, CVDs caused 7.08 million deaths worldwide, surging from 6.6 million deaths in 2019.^116^, ^163^ Recent studies have elucidated the pivotal roles of UMEs in CVDs, shedding light on the mechanism and therapy of these medical emergencies. Despite these advances, several critical aspects concerning UMEs in CVDs remain to be strengthened. First, more effects are needed to delineate the disease linkage of UMEs with CVDs. Inflammation is of particular importance in the progression of CVDs. Some UMEs, such as Pellino, are impactful regulators of inflammatory signaling, but their roles in CVDs remain largely unknown. Besides, no UME has been found to regulate cerebral small vessel diseases to date. It is intriguing and meaningful to identify new CVD‐regulating UMEs. Second, the function of some UMEs in CVDs has yet to be clarified. Although RNF213 has been established as an essential protein in MMD, its exact function in MMD remains unclear. In the future, the in‐depth investigation of new biochemical functions, interacting partners, and substrates of RNF213 may unravel the pathogenic mechanisms of MMD and inspire new therapies for MMD. Third, the clinical relevance of UMEs with human CVDs should be confirmed. Given that animal models cannot fully recapitulate human diseases and most studies have explored the function of UMEs in CVDs using animal models, these findings cannot be simply extrapolated to clinical situations. Comprehensive studies involving clinical research or humanized mice are more favorable for concluding the function of UMEs in CVDs. Fourth, the research and development of therapeutic approaches and drugs for CVDs based on UMEs should be accelerated. Despite recent advances, the study on UME inhibitors/agonists and PROTAC/DUBTAC is still in its infancy. Further studies in this burgeoning field may improve or even revolutionize the treatment for CVDs.

## AUTHOR CONTRIBUTIONS

*Conceptualization*: Xu Wang. *Data collection and analysis*: Jingyong Huang, Zhenhu Zhu, and Xu Wang. *Writing‐original draft*: Jingyong Huang, Zhenhu Zhu, and Xu Wang. *Writing‐review and editing*: Jingyong Huang, Zhenhu Zhu, Dirk Schlüter, Kate Lykke Lambertsen, Weihong Song, and Xu Wang. *Funding acquisition*: Weihong Song and Xu Wang.

## CONFLICT OF INTEREST STATEMENT

The authors declare no conflicts of interest.

## ETHICAL APPROVAL

Not applicable.

## Data Availability

Not applicable.

## References

[1] Mergenthaler P , Lindauer U , Dienel GA , Meisel A . Sugar for the brain: The role of glucose in physiological and pathological brain function. Trends Neurosci. 2013;36(10):587‐597. doi:10.1016/j.tins.2013.07.001 23968694 PMC3900881

[2] Belanger M , Allaman I , Magistretti PJ . Brain energy metabolism: Focus on astrocyte‐neuron metabolic cooperation. Cell Metab. 2011;14(6):724‐738. doi:10.1016/j.cmet.2011.08.016 22152301

[3] Xing CY , Tarumi T , Liu J , et al. Distribution of cardiac output to the brain across the adult lifespan. J Cereb Blood Flow Metab. 2017;37(8):2848‐2856. doi:10.1177/0271678x16676826 27789785 PMC5536794

[4] Claassen J , Thijssen DHJ , Panerai RB , Faraci FM . Regulation of cerebral blood flow in humans: physiology and clinical implications of autoregulation. Physiol Rev. 2021;101(4):1487‐1559. doi:10.1152/physrev.00022.2020 33769101 PMC8576366

[5] Gutierrez J , Khasiyev F , Liu M , et al. Determinants and outcomes of asymptomatic intracranial atherosclerotic stenosis. J Am Coll Cardiol. 2021;78(6):562‐571. doi:10.1016/j.jacc.2021.05.041 34353533 PMC8352282

[6] Banerjee C , Chimowitz MI . Stroke caused by atherosclerosis of the major intracranial arteries. Circ Res. 2017;120(3):502‐513. doi:10.1161/CIRCRESAHA.116.308441 28154100 PMC5312775

[7] Ihara M , Yamamoto Y , Hattori Y , et al. Moyamoya disease: diagnosis and interventions. Lancet Neurol. 2022;21(8):747‐758. doi:10.1016/S1474-4422(22)00165-X 35605621

[8] Asselman C , Hemelsoet D , Eggermont D , Dermaut B , Impens F . Moyamoya disease emerging as an immune‐related angiopathy. Trends Mol Med. 2022;28(11):939‐950. doi:10.1016/j.molmed.2022.08.009 36115805

[9] Tekle WG , Hassan AE . Intracranial Atherosclerotic disease: current concepts in medical and surgical management. Neurology. 2021;97(20):S145‐S157. doi:10.1212/WNL.0000000000012805 Suppl 2 34785613

[10] Owolabi MO , Thrift AG , Mahal A , et al. Primary stroke prevention worldwide: translating evidence into action. Lancet Public Health. 2022;7(1):e74‐e85. doi:10.1016/S2468-2667(21)00230-9 34756176 PMC8727355

[11] Liu H , Sun S , Liu B . Smurf2 exerts neuroprotective effects on cerebral ischemic injury. J Biol Chem. 2021;297(2):100537. doi:10.1016/j.jbc.2021.100537 33722608 PMC8363835

[12] Yu YL , Chou RH , Shyu WC , et al. Smurf2‐mediated degradation of EZH2 enhances neuron differentiation and improves functional recovery after ischaemic stroke. EMBO Mol Med. 2013;5(4):531‐547. doi:10.1002/emmm.201201783 23526793 PMC3628108

[13] Zeng J , Wang Y , Luo Z , et al. TRIM9‐Mediated resolution of neuroinflammation confers neuroprotection upon ischemic stroke in mice. Cell Rep. 2019;27(2):549‐560. doi:10.1016/j.celrep.2018.12.055 e6 30970257 PMC6485958

[14] Liu H , Povysheva N , Rose ME , et al. Role of UCHL1 in axonal injury and functional recovery after cerebral ischemia. Proc Natl Acad Sci U S A. 2019;116(10):4643‐4650. doi:10.1073/pnas.1821282116 30760601 PMC6410860

[15] Roy V , Ross JP , Pepin R , et al. Moyamoya disease susceptibility gene RNF213 regulates endothelial barrier function. Stroke. 2022;53(4):1263‐1275. doi:10.1161/STROKEAHA.120.032691 34991336

[16] Ndoja A , Reja R , Lee SH , et al. Ubiquitin ligase COP1 suppresses neuroinflammation by degrading c/EBPbeta in microglia. Cell. 2020;182(5):1156‐1169. doi:10.1016/j.cell.2020.07.011 e12 32795415

[17] Heger K , Wickliffe KE , Ndoja A , et al. OTULIN limits cell death and inflammation by deubiquitinating LUBAC. Nature. 2018;559(7712):120‐124. doi:10.1038/s41586-018-0256-2 29950720

[18] Zheng N , Shabek N . Ubiquitin ligases: Structure, function, and regulation. Annu Rev Biochem. 2017;86:129‐157. doi:10.1146/annurev-biochem-060815-014922 28375744

[19] Berndsen CE , Wolberger C . New insights into ubiquitin E3 ligase mechanism. Nat Struct Mol Biol. 2014;21(4):301‐307. doi:10.1038/nsmb.2780 24699078

[20] Harrigan JA , Jacq X , Martin NM , Jackson SP . Deubiquitylating enzymes and drug discovery: Emerging opportunities. Nat Rev Drug Discovery. 2018;17(1):57‐78. doi:10.1038/nrd.2017.152 28959952 PMC7097658

[21] Deol KK , Lorenz S , Strieter ER . Enzymatic logic of ubiquitin chain assembly. Front Physiol. 2019;10:835. doi:10.3389/fphys.2019.00835 31333493 PMC6624479

[22] Clague MJ , Urbe S , Komander D . Breaking the chains: deubiquitylating enzyme specificity begets function. Nat Rev Mol Cell Biol. 2019;20(6):338‐352. doi:10.1038/s41580-019-0099-1 30733604

[23] Liu B , Ruan J , Chen M , et al. Deubiquitinating enzymes (DUBs): Decipher underlying basis of neurodegenerative diseases. Mol Psychiatry. 2022;27(1):259‐268. doi:10.1038/s41380-021-01233-8 34285347

[24] Juang YC , Landry MC , Sanches M , et al. OTUB1 co‐opts Lys48‐linked ubiquitin recognition to suppress E2 enzyme function. Mol Cell. 2012;45(3):384‐397. doi:10.1016/j.molcel.2012.01.011 22325355 PMC3306812

[25] Wiener R , Zhang X , Wang T , Wolberger C . The mechanism of OTUB1‐mediated inhibition of ubiquitination. Nature. 2012;483(7391):618‐622. doi:10.1038/nature10911 22367539 PMC3319311

[26] Nakada S , Tai I , Panier S , et al. Non‐canonical inhibition of DNA damage‐dependent ubiquitination by OTUB1. Nature. 2010;466(7309):941‐946. doi:10.1038/nature09297 20725033

[27] Sheng X , Xia Z , Yang H , Hu R . The ubiquitin codes in cellular stress responses. Protein Cell. 2023;15(3):157‐190. doi:10.1093/procel/pwad045 PMC10903993 37470788

[28] Gu W , Zhang J , Li Q , et al. The TRIM37 variants in Mulibrey nanism patients paralyze follicular helper T cell differentiation. Cell Discovery. 2023;9(1):82. doi:10.1038/s41421-023-00561-z 37528081 PMC10394018

[29] Brigant B , Demont Y , Ouled‐Haddou H , et al. TRIM37 is highly expressed during mitosis in CHON‐002 chondrocytes cell line and is regulated by miR‐223. Bone. 2020;137:115393. doi:10.1016/j.bone.2020.115393 32353567

[30] Liu W , Morito D , Takashima S , et al. Identification of RNF213 as a susceptibility gene for moyamoya disease and its possible role in vascular development. PLoS One. 2011;6(7):e22542. doi:10.1371/journal.pone.0022542 21799892 PMC3140517

[31] Kamada F , Aoki Y , Narisawa A , et al. A genome‐wide association study identifies RNF213 as the first Moyamoya disease gene. J Hum Genet. 2011;56(1):34‐40. doi:10.1038/jhg.2010.132 21048783

[32] Liu W , Hitomi T , Kobayashi H , Harada KH , Koizumi A . Distribution of moyamoya disease susceptibility polymorphism p.R4810K in RNF213 in East and Southeast Asian populations. Neurol Med Chir. 2012;52(5):299‐303. doi:10.2176/nmc.52.299 22688066

[33] Jang MA , Shin S , Yoon JH , Ki CS . Frequency of the moyamoya‐related RNF213 p.Arg4810Lys variant in 1,516 Korean individuals. BMC Med Genet. 2015;16:109. doi:10.1186/s12881-015-0252-4 26590131 PMC4654917

[34] Xue Y , Zeng C , Ge P , et al. Association of RNF213 variants with periventricular anastomosis in moyamoya disease. Stroke. 2022;53(9):2906‐2916. doi:10.1161/STROKEAHA.121.038066 35543128

[35] Collaborators GBDS . Global, regional, and national burden of stroke, 1990–2016: A systematic analysis for the global burden of disease study 2016. Lancet Neurol. 2019;18(5):439‐458. doi:10.1016/S1474-4422(19)30034-1 30871944 PMC6494974

[36] Hankey GJ . Stroke. Lancet. 2017;389(10069):641‐654. doi:10.1016/S0140-6736(16)30962-X 27637676

[37] Prabhakaran S , Ruff I , Bernstein RA . Acute stroke intervention: a systematic review. JAMA. 2015;313(14):1451‐1462. doi:10.1001/jama.2015.3058 25871671

[38] Bavarsad K , Barreto GE , Hadjzadeh MA , Sahebkar A . Protective effects of curcumin against ischemia‐reperfusion injury in the nervous system. Mol Neurobiol. 2019;56(2):1391‐1404. doi:10.1007/s12035-018-1169-7 29948942

[39] Naito MG , Xu D , Amin P , et al. Sequential activation of necroptosis and apoptosis cooperates to mediate vascular and neural pathology in stroke. Proc Natl Acad Sci U S A. 2020;117(9):4959‐4970. doi:10.1073/pnas.1916427117 32071228 PMC7060720

[40] Green DR . The coming decade of cell death research: five riddles. Cell. 2019;177(5):1094‐1107. doi:10.1016/j.cell.2019.04.024 31100266 PMC6534278

[41] Galluzzi L , Kepp O , Chan FK , Kroemer G . Necroptosis: mechanisms and relevance to disease. Annu Rev Pathol. 2017;12:103‐130. doi:10.1146/annurev-pathol-052016-100247 27959630 PMC5786374

[42] Yuan J , Amin P , Ofengeim D . Necroptosis and RIPK1‐mediated neuroinflammation in CNS diseases. Nat Rev Neurosci. 2019;20(1):19‐33. doi:10.1038/s41583-018-0093-1 30467385 PMC6342007

[43] Pasparakis M , Vandenabeele P . Necroptosis and its role in inflammation. Nature. 2015;517(7534):311‐320. doi:10.1038/nature14191 25592536

[44] Deng XX , Li SS , Sun FY . Necrostatin‐1 Prevents necroptosis in brains after ischemic stroke via inhibition of RIPK1‐mediated RIPK3/MLKL signaling. Aging Dis. 2019;10(4):807‐817. doi:10.14336/AD.2018.0728 31440386 PMC6675533

[45] Degterev A , Huang Z , Boyce M , et al. Chemical inhibitor of nonapoptotic cell death with therapeutic potential for ischemic brain injury. Nat Chem Biol. 2005;1(2):112‐119. doi:10.1038/nchembio711 16408008

[46] Li J , Zhang J , Zhang Y , et al. TRAF2 protects against cerebral ischemia‐induced brain injury by suppressing necroptosis. Cell Death Dis. 2019;10(5):328. doi:10.1038/s41419-019-1558-5 30988281 PMC6465397

[47] Yao D , Zhang S , Hu Z , et al. CHIP ameliorates cerebral ischemia‐reperfusion injury by attenuating necroptosis and inflammation. Aging (Albany NY). 2021;13(23):25564‐25577. doi:10.18632/aging.203774 34905731 PMC8714161

[48] Yuan Z , Yi‐Yun S , Hai‐Yan Y . Triad3A displays a critical role in suppression of cerebral ischemic/reperfusion (I/R) injury by regulating necroptosis. Biomed Pharmacother. 2020;128:110045. doi:10.1016/j.biopha.2020.110045 32460187

[49] Cabral‐Miranda F , Nicoloso‐Simoes E , Adao‐Novaes J , et al. rAAV8‐733‐Mediated gene transfer of CHIP/Stub‐1 prevents hippocampal neuronal death in experimental brain ischemia. Mol Ther. 2017;25(2):392‐400. doi:10.1016/j.ymthe.2016.11.017 28153090 PMC5368595

[50] Rodriguez C , Ramos‐Araque ME , Dominguez‐Martinez M , et al. Single‐nucleotide polymorphism 309T>G in the MDM2 promoter determines functional outcome after stroke. Stroke. 2018;49(10):2437‐2444. doi:10.1161/STROKEAHA.118.022529 30355102 PMC6159670

[51] Zhu X , Li J , You D , Xiao Y , Huang Z , Yu W . Neuroprotective effect of E3 ubiquitin ligase RNF8 against ischemic stroke via HDAC2 stability reduction and reelin‐dependent GSK3beta inhibition. Mol Neurobiol. 2022;59(8):4776‐4790. doi:10.1007/s12035-022-02880-w 35622272 PMC9135995

[52] Gu C , Yang J , Luo Y , et al. ZNRF2 attenuates focal cerebral ischemia/reperfusion injury in rats by inhibiting mTORC1‐mediated autophagy. Exp Neurol. 2021;342:113759. doi:10.1016/j.expneurol.2021.113759 33992580

[53] Wang L , Lu Y , Guan H , et al. Tumor necrosis factor receptor‐associated factor 5 is an essential mediator of ischemic brain infarction. J Neurochem. 2013;126(3):400‐414. doi:10.1111/jnc.12207 23413803

[54] Li T , Qin JJ , Yang X , et al. The Ubiquitin E3 Ligase TRAF6 exacerbates ischemic stroke by ubiquitinating and activating Rac1. J Neurosci. 2017;37(50):12123‐12140. doi:10.1523/JNEUROSCI.1751-17.2017 29114077 PMC6596816

[55] Gong J , Li ZZ , Guo S , et al. Neuron‐Specific tumor necrosis factor receptor‐associated factor 3 is a central regulator of neuronal death in acute ischemic stroke. Hypertension. 2015;66(3):604‐616. doi:10.1161/HYPERTENSIONAHA.115.05430 26269654

[56] Gomez‐Sanchez JC , Delgado‐Esteban M , Rodriguez‐Hernandez I , et al. The human Tp53 Arg72Pro polymorphism explains different functional prognosis in stroke. J Exp Med. 2011;208(3):429‐437. doi:10.1084/jem.20101523 21357744 PMC3058581

[57] Rodriguez C , Sobrino T , Agulla J , et al. Neovascularization and functional recovery after intracerebral hemorrhage is conditioned by the Tp53 Arg72Pro single‐nucleotide polymorphism. Cell Death Differ. 2017;24(1):144‐154. doi:10.1038/cdd.2016.109 27768124 PMC5260494

[58] Ouyang S , Song Y , Tian Y , Chen Y , Yu X , Wang D . RNF8 deficiency results in neurodegeneration in mice. Neurobiol Aging. 2015;36(10):2850‐2860. doi:10.1016/j.neurobiolaging.2015.07.010 26256786 PMC4851709

[59] Mailand N , Bekker‐Jensen S , Faustrup H , et al. RNF8 ubiquitylates histones at DNA double‐strand breaks and promotes assembly of repair proteins. Cell. 2007;131(5):887‐900. doi:10.1016/j.cell.2007.09.040 18001824

[60] Wang Y , Liu G , Hong D , Chen F , Ji X , Cao G . White matter injury in ischemic stroke. Prog Neurobiol. 2016;141:45‐60. doi:10.1016/j.pneurobio.2016.04.005 27090751 PMC5677601

[61] Stetler RA , Gao Y , Leak RK , et al. APE1/Ref‐1 facilitates recovery of gray and white matter and neurological function after mild stroke injury. Proc Natl Acad Sci U S A. 2016;113(25):E3558‐E3567. doi:10.1073/pnas.1606226113 27274063 PMC4922172

[62] Liu H , Li W , Rose ME , et al. The point mutation UCH‐L1 C152A protects primary neurons against cyclopentenone prostaglandin‐induced cytotoxicity: implications for post‐ischemic neuronal injury. Cell Death Dis. 2015;6(11):e1966. doi:10.1038/cddis.2015.323 26539913 PMC4670930

[63] Shichita T , Ooboshi H , Yoshimura A . Neuroimmune mechanisms and therapies mediating post‐ischaemic brain injury and repair. Nat Rev Neurosci. 2023;24(5):299‐312. doi:10.1038/s41583-023-00690-0 36973481

[64] Heckmann BL , Tummers B , Green DR . Crashing the computer: apoptosis vs. necroptosis in neuroinflammation. Cell Death Differ. 2019;26(1):41‐52. doi:10.1038/s41418-018-0195-3 30341422 PMC6294765

[65] Xiao Y , Jin J , Chang M , et al. Peli1 promotes microglia‐mediated CNS inflammation by regulating Traf3 degradation. Nat Med. 2013;19(5):595‐602. doi:10.1038/nm.3111 23603814 PMC3899792

[66] Wang X , Deckert M , Xuan NT , et al. Astrocytic A20 ameliorates experimental autoimmune encephalomyelitis by inhibiting NF‐kappaB‐ and STAT1‐dependent chemokine production in astrocytes. Acta Neuropathol. 2013;126(5):711‐724. doi:10.1007/s00401-013-1183-9 24077734

[67] Mulas F , Wang X , Song S , et al. The deubiquitinase OTUB1 augments NF‐kappaB‐dependent immune responses in dendritic cells in infection and inflammation by stabilizing UBC13. Cell Mol Immunol. 2021;18(6):1512‐1527. doi:10.1038/s41423-020-0362-6 32024978 PMC8167118

[68] Wang X , Mulas F , Yi W , et al. OTUB1 inhibits CNS autoimmunity by preventing IFN‐gamma‐induced hyperactivation of astrocytes. EMBO J. 2019;38(10):e100947. doi:10.15252/embj.2018100947 30944096 PMC6517825

[69] Cruz JA , Mokashi CS , Kowalczyk GJ , et al. A variable‐gain stochastic pooling motif mediates information transfer from receptor assemblies into NF‐kappaB. Sci Adv. 2021;7(30):eabi9410. doi:10.1126/sciadv.abi9410 34301608 PMC8302133

[70] Tarantino N , Tinevez JY , Crowell EF , et al. TNF and IL‐1 exhibit distinct ubiquitin requirements for inducing NEMO‐IKK supramolecular structures. J Cell Biol. 2014;204(2):231‐245. doi:10.1083/jcb.201307172 24446482 PMC3897181

[71] Bai X , Zhang YL , Liu LN . Inhibition of TRIM8 restrains ischaemia‐reperfusion‐mediated cerebral injury by regulation of NF‐kappaB activation associated inflammation and apoptosis. Exp Cell Res. 2020;388(2):111818. doi:10.1016/j.yexcr.2020.111818 31917201

[72] Xia Q , Zhan G , Mao M , Zhao Y , Li X . TRIM45 causes neuronal damage by aggravating microglia‐mediated neuroinflammation upon cerebral ischemia and reperfusion injury. Exp Mol Med. 2022;54(2):180‐193. doi:10.1038/s12276-022-00734-y 35217833 PMC8894463

[73] Hao MQ , Xie LJ , Leng W , Xue RW . Trim47 is a critical regulator of cerebral ischemia‐reperfusion injury through regulating apoptosis and inflammation. Biochem Biophys Res Commun. 2019;515(4):651‐657. doi:10.1016/j.bbrc.2019.05.065 31178138

[74] Liu X , Lei Q . TRIM62 knockout protects against cerebral ischemic injury in mice by suppressing NLRP3‐regulated neuroinflammation. Biochem Biophys Res Commun. 2020;529(2):140‐147. doi:10.1016/j.bbrc.2020.06.014 32703402

[75] Wang L , Wu D , Xu Z . USP10 protects against cerebral ischemia injury by suppressing inflammation and apoptosis through the inhibition of TAK1 signaling. Biochem Biophys Res Commun. 2019;516(4):1272‐1278. doi:10.1016/j.bbrc.2019.06.042 31301769

[76] Xiang J , Zhang X , Fu J , Wang H , Zhao Y . USP18 overexpression protects against focal cerebral ischemia injury in mice by suppressing microglial activation. Neuroscience. 2019;419:121‐128. doi:10.1016/j.neuroscience.2019.09.001 31513843

[77] Pan R , Xie Y , Fang W , Liu Y , Zhang Y . USP20 mitigates ischemic stroke in mice by suppressing neuroinflammation and neuron death via regulating PTEN signal. Int Immunopharmacol. 2022;103:107840. doi:10.1016/j.intimp.2021.107840 34953448

[78] Li Z , Liu B , Lambertsen KL , et al. USP25 inhibits neuroinflammatory responses after cerebral ischemic stroke by deubiquitinating TAB2. Adv Sci. 2023;10(28):e2301641. doi:10.1002/advs.202301641 PMC10558664 37587766

[79] Frescas D , Pagano M . Deregulated proteolysis by the F‐box proteins SKP2 and beta‐TrCP: tipping the scales of cancer. Nat Rev Cancer. 2008;8(6):438‐449. doi:10.1038/nrc2396 18500245 PMC2711846

[80] Shi M , Cho H , Inn KS , et al. Negative regulation of NF‐kappaB activity by brain‐specific TRIpartite Motif protein 9. Nat Commun. 2014;5:4820. doi:10.1038/ncomms5820 25190485 PMC4157316

[81] Spitzer D , Guerit S , Puetz T , et al. Profiling the neurovascular unit unveils detrimental effects of osteopontin on the blood‐brain barrier in acute ischemic stroke. Acta Neuropathol. 2022;144(2):305‐337. doi:10.1007/s00401-022-02452-1 35752654 PMC9288377

[82] Yu H , Luo H , Chang L , et al. The NEDD8‐activating enzyme inhibitor MLN4924 reduces ischemic brain injury in mice. Proc Natl Acad Sci U S A. 2022;119(6):e2111896119. doi:10.1073/pnas.2111896119 35101976 PMC8833173

[83] Narne P , Pandey V , Phanithi PB . Interplay between mitochondrial metabolism and oxidative stress in ischemic stroke: an epigenetic connection. Mol Cell Neurosci. 2017;82:176‐194. doi:10.1016/j.mcn.2017.05.008 28552342

[84] Ren KD , Liu WN , Tian J , et al. Mitochondrial E3 ubiquitin ligase 1 promotes brain injury by disturbing mitochondrial dynamics in a rat model of ischemic stroke. Eur J Pharmacol. 2019;861:172617. doi:10.1016/j.ejphar.2019.172617 31430457

[85] Prudent J , Zunino R , Sugiura A , Mattie S , Shore GC , McBride HM . MAPL SUMOylation of Drp1 stabilizes an ER/mitochondrial platform required for cell death. Mol Cell. 2015;59(6):941‐955. doi:10.1016/j.molcel.2015.08.001 26384664

[86] Sisalli MJ , Ianniello G , Savoia C , Cuomo O , Annunziato L , Scorziello A . Knocking‐out the Siah2 E3 ubiquitin ligase prevents mitochondrial NCX3 degradation, regulates mitochondrial fission and fusion, and restores mitochondrial function in hypoxic neurons. Cell Commun Signal. 2020;18(1):42. doi:10.1186/s12964-020-0529-x 32164721 PMC7066748

[87] Carlucci A , Adornetto A , Scorziello A , et al. Proteolysis of AKAP121 regulates mitochondrial activity during cellular hypoxia and brain ischaemia. EMBO J. 2008;27(7):1073‐1084. doi:10.1038/emboj.2008.33 18323779 PMC2323260

[88] Zhang X , Yan H , Yuan Y , et al. Cerebral ischemia‐reperfusion‐induced autophagy protects against neuronal injury by mitochondrial clearance. Autophagy. 2013;9(9):1321‐1333. doi:10.4161/auto.25132 23800795

[89] Geisler S , Holmstrom KM , Skujat D , et al. PINK1/Parkin‐mediated mitophagy is dependent on VDAC1 and p62/SQSTM1. Nat Cell Biol. 2010;12(2):119‐131. doi:10.1038/ncb2012 20098416

[90] Zhang X , Yuan Y , Jiang L , et al. Endoplasmic reticulum stress induced by tunicamycin and thapsigargin protects against transient ischemic brain injury: Involvement of PARK2‐dependent mitophagy. Autophagy. 2014;10(10):1801‐1813. doi:10.4161/auto.32136 25126734 PMC4198364

[91] Feigin VL , Lawes CM , Bennett DA , Barker‐Collo SL , Parag V . Worldwide stroke incidence and early case fatality reported in 56 population‐based studies: A systematic review. Lancet Neurol. 2009;8(4):355‐369. doi:10.1016/S1474-4422(09)70025-0 19233729

[92] Claassen J , Park S . Spontaneous subarachnoid haemorrhage. Lancet. 2022;400(10355):846‐862. doi:10.1016/S0140-6736(22)00938-2 35985353 PMC9987649

[93] Aronowski J , Zhao X . Molecular pathophysiology of cerebral hemorrhage: secondary brain injury. Stroke. 2011;42(6):1781‐1786. doi:10.1161/STROKEAHA.110.596718 21527759 PMC3123894

[94] Meng Z , Zhao T , Zhou K , et al. A20 Ameliorates intracerebral hemorrhage‐induced inflammatory injury by regulating TRAF6 polyubiquitination. J Immunol. 2017;198(2):820‐831. doi:10.4049/jimmunol.1600334 27986908 PMC5220121

[95] Qu X , Wang N , Chen W , Qi M , Xue Y , Cheng W . RNF34 overexpression exacerbates neurological deficits and brain injury in a mouse model of intracerebral hemorrhage by potentiating mitochondrial dysfunction‐mediated oxidative stress. Sci Rep. 2019;9(1):16296. doi:10.1038/s41598-019-52494-x 31704983 PMC6841714

[96] Zhang S , Hu ZW , Luo HY , et al. AAV/BBB‐mediated gene transfer of CHIP attenuates brain injury following experimental intracerebral hemorrhage. Transl Stroke Res. 2020;11(2):296‐309. doi:10.1007/s12975-019-00715-w 31325153

[97] Liu C , Liu C , Liu H , et al. Increased expression of ubiquitin‐specific protease 4 participates in neuronal apoptosis after intracerebral hemorrhage in adult rats. Cell Mol Neurobiol. 2017;37(3):427‐435. doi:10.1007/s10571-016-0375-y 27114249 PMC11482130

[98] Xu Z , Li X , Chen J , et al. USP11, Deubiquitinating enzyme, associated with neuronal apoptosis following intracerebral hemorrhage. J Mol Neurosci. 2016;58(1):16‐27. doi:10.1007/s12031-015-0644-0 26334325

[99] Ma A , Malynn BA . A20: linking a complex regulator of ubiquitylation to immunity and human disease. Nat Rev Immunol. 2012;12(11):774‐785. doi:10.1038/nri3313 23059429 PMC3582397

[100] Ruan J , Schluter D , Naumann M , Waisman A , Wang X . Ubiquitin‐modifying enzymes as regulators of colitis. Trends Mol Med. 2022;28(4):304‐318. doi:10.1016/j.molmed.2022.01.006 35177326

[101] Razani B , Whang MI , Kim FS , et al. Non‐catalytic ubiquitin binding by A20 prevents psoriatic arthritis‐like disease and inflammation. Nat Immunol. 2020;21(4):422‐433. doi:10.1038/s41590-020-0634-4 32205880 PMC7195210

[102] St‐Pierre J , Drori S , Uldry M , et al. Suppression of reactive oxygen species and neurodegeneration by the PGC‐1 transcriptional coactivators. Cell. 2006;127(2):397‐408. doi:10.1016/j.cell.2006.09.024 17055439

[103] Chu X , Wu X , Feng H , et al. Coupling between interleukin‐1R1 and necrosome complex involves in hemin‐induced neuronal necroptosis after intracranial hemorrhage. Stroke. 2018;49(10):2473‐2482. doi:10.1161/STROKEAHA.117.019253 30355103

[104] Shen H , Liu C , Zhang D , et al. Role for RIP1 in mediating necroptosis in experimental intracerebral hemorrhage model both in vivo and in vitro. Cell Death Dis. 2017;8(3):e2641. doi:10.1038/cddis.2017.58 28252651 PMC5386555

[105] Seo J , Lee EW , Sung H , et al. CHIP controls necroptosis through ubiquitylation‐ and lysosome‐dependent degradation of RIPK3. Nat Cell Biol. 2016;18(3):291‐302. doi:10.1038/ncb3314 26900751

[106] Chen S , Feng H , Sherchan P , et al. Controversies and evolving new mechanisms in subarachnoid hemorrhage. Prog Neurobiol. 2014;115:64‐91. doi:10.1016/j.pneurobio.2013.09.002 24076160 PMC3961493

[107] Mabb AM , Je HS , Wall MJ , et al. Triad3A regulates synaptic strength by ubiquitination of arc. Neuron. 2014;82(6):1299‐1316. doi:10.1016/j.neuron.2014.05.016 24945773 PMC4277707

[108] Chen T , Zhu J , Wang YH . RNF216 mediates neuronal injury following experimental subarachnoid hemorrhage through the Arc/Arg3.1‐AMPAR pathway. FASEB J. 2020;34(11):15080‐15092. doi:10.1096/fj.201903151RRRR 32918771

[109] Zhou Y , Tao T , Liu G , et al. TRAF3 mediates neuronal apoptosis in early brain injury following subarachnoid hemorrhage via targeting TAK1‐dependent MAPKs and NF‐kappaB pathways. Cell Death Dis. 2021;12(1):10. doi:10.1038/s41419-020-03278-z 33414375 PMC7790824

[110] Huang XP , Peng JH , Pang JW , et al. Peli1 Contributions in microglial activation, neuroinflammatory responses and neurological deficits following experimental subarachnoid hemorrhage. Front Mol Neurosci. 2017;10:398. doi:10.3389/fnmol.2017.00398 29249938 PMC5714869

[111] Scott RM , Smith ER . Moyamoya disease and moyamoya syndrome. N Engl J Med. 2009;360(12):1226‐1237. doi:10.1056/NEJMra0804622 19297575

[112] Kuroda S , Houkin K . Moyamoya disease: Current concepts and future perspectives. Lancet Neurol. 2008;7(11):1056‐1066. doi:10.1016/S1474-4422(08)70240-0 18940695

[113] Ahel J , Lehner A , Vogel A , et al. Moyamoya disease factor RNF213 is a giant E3 ligase with a dynein‐like core and a distinct ubiquitin‐transfer mechanism. eLife. 2020;9:e56185. doi:10.7554/eLife.56185 32573437 PMC7311170

[114] Bhardwaj A , Banh RS , Zhang W , Sidhu SS , Neel BG . MMD‐associated RNF213 SNPs encode dominant‐negative alleles that globally impair ubiquitylation. Life Sci Alliance. 2022;5(5):e202000807. doi:10.26508/lsa.202000807 35135845 PMC8831215

[115] Wolf D , Ley K . Immunity and inflammation in atherosclerosis. Circ Res. 2019;124(2):315‐327. doi:10.1161/CIRCRESAHA.118.313591 30653442 PMC6342482

[116] Tsao CW , Aday AW , Almarzooq ZI , et al. Heart disease and stroke statistics‐2022 update: A report from the American Heart Association. Circulation. 2022;145(8):e153‐e639. doi:10.1161/CIR.0000000000001052 35078371

[117] Qureshi AI , Caplan LR . Intracranial atherosclerosis. Lancet. 2014;383(9921):984‐998. doi:10.1016/S0140-6736(13)61088-0 24007975

[118] Bang OY , Chung JW , Cha J , et al. A polymorphism in RNF213 is a susceptibility gene for intracranial atherosclerosis. PLoS One. 2016;11(6):e0156607. doi:10.1371/journal.pone.0156607 27253870 PMC4890790

[119] Okazaki S , Morimoto T , Kamatani Y , et al. Moyamoya disease susceptibility variant RNF213 p.R4810K Increases the risk of ischemic stroke attributable to large‐artery atherosclerosis. Circulation. 2019;139(2):295‐298. doi:10.1161/CIRCULATIONAHA.118.038439 30615506

[120] Kamimura T , Okazaki S , Morimoto T , et al. Prevalence of RNF213 p.R4810K variant in early‐onset stroke with intracranial arterial stenosis. Stroke. 2019;50(6):1561‐1563. doi:10.1161/STROKEAHA.118.024712 31060437

[121] Miyawaki S , Imai H , Shimizu M , et al. Genetic variant RNF213 c.14576G>A in various phenotypes of intracranial major artery stenosis/occlusion. Stroke. 2013;44(10):2894‐2897. doi:10.1161/STROKEAHA.113.002477 23970789

[122] Kim HJ , Choi EH , Chung JW , et al. Role of the RNF213 variant in vascular outcomes in patients with intracranial atherosclerosis. J Am Heart Assoc. 2021;10(1):e017660. doi:10.1161/JAHA.120.017660 33356381 PMC7955464

[123] Wang X , Wang Y , Antony V , Sun H , Liang G . Metabolism‐associated molecular patterns (MAMPs). Trends Endocrinol Metab. 2020;31(10):712‐724. doi:10.1016/j.tem.2020.07.001 32807598

[124] Kunjathoor VV , Febbraio M , Podrez EA , et al. Scavenger receptors class A‐I/II and CD36 are the principal receptors responsible for the uptake of modified low density lipoprotein leading to lipid loading in macrophages. J Biol Chem. 2002;277(51):49982‐49988. doi:10.1074/jbc.M209649200 12376530

[125] Chinetti‐Gbaguidi G , Colin S , Staels B . Macrophage subsets in atherosclerosis. Nat Rev Cardiol. 2015;12(1):10‐17. doi:10.1038/nrcardio.2014.173 25367649

[126] Shankman LS , Gomez D , Cherepanova OA , et al. KLF4‐dependent phenotypic modulation of smooth muscle cells has a key role in atherosclerotic plaque pathogenesis. Nat Med. 2015;21(6):628‐637. doi:10.1038/nm.3866 25985364 PMC4552085

[127] Skalen K , Gustafsson M , Rydberg EK , et al. Subendothelial retention of atherogenic lipoproteins in early atherosclerosis. Nature. 2002;417(6890):750‐754. doi:10.1038/nature00804 12066187

[128] Li Q , Xuan W , Jia Z , et al. HRD1 prevents atherosclerosis‐mediated endothelial cell apoptosis by promoting LOX‐1 degradation. Cell Cycle. 2020;19(12):1466‐1477. doi:10.1080/15384101.2020.1754561 32308114 PMC7469520

[129] Zeng Y , Cao J , Li CX , Wang CY , Wu RM , Xu XL . MDM2‐mediated ubiquitination of RXRbeta contributes to mitochondrial damage and related inflammation in atherosclerosis. Int J Mol Sci. 2022;23(10):5766. doi:10.3390/ijms23105766 35628577 PMC9145909

[130] Wang X , Ma L , Zhang S , Song Q , He X , Wang J . WWP2 ameliorates oxidative stress and inflammation in atherosclerotic mice through regulation of PDCD4/HO‐1 pathway. Acta Biochim Biophys Sin. 2022;54(8):1057‐1067. doi:10.3724/abbs.2022091 35983977 PMC9828489

[131] Akhmedov A , Sawamura T , Chen CH , Kraler S , Vdovenko D , Luscher TF . Lectin‐like oxidized low‐density lipoprotein receptor‐1 (LOX‐1): a crucial driver of atherosclerotic cardiovascular disease. Eur Heart J. 2021;42(18):1797‐1807. doi:10.1093/eurheartj/ehaa770 33159784

[132] Rahaman SO , Lennon DJ , Febbraio M , Podrez EA , Hazen SL , Silverstein RL . A CD36‐dependent signaling cascade is necessary for macrophage foam cell formation. Cell Metab. 2006;4(3):211‐221. doi:10.1016/j.cmet.2006.06.007 16950138 PMC1855263

[133] Wang B , Tang X , Yao L , et al. Disruption of USP9X in macrophages promotes foam cell formation and atherosclerosis. J Clin Invest. 2022;132(10):e154217. doi:10.1172/JCI154217 35389885 PMC9106359

[134] Tran TT , Poirier H , Clement L , et al. Luminal lipid regulates CD36 levels and downstream signaling to stimulate chylomicron synthesis. J Biol Chem. 2011;286(28):25201‐25210. doi:10.1074/jbc.M111.233551 21610069 PMC3137091

[135] Srikanthan S , Li W , Silverstein RL , McIntyre TM . Exosome poly‐ubiquitin inhibits platelet activation, downregulates CD36 and inhibits pro‐atherothombotic cellular functions. J Thromb Haemost. 2014;12(11):1906‐1917. doi:10.1111/jth.12712 25163645 PMC4229405

[136] Xia X , Hu T , He J , et al. USP10 deletion inhibits macrophage‐derived foam cell formation and cellular‐oxidized low density lipoprotein uptake by promoting the degradation of CD36. Aging. 2020;12(22):22892‐22905. doi:10.18632/aging.104003 33197885 PMC7746336

[137] Zhang F , Xia X , Chai R , et al. Inhibition of USP14 suppresses the formation of foam cell by promoting CD36 degradation. J Cell Mol Med. 2020;24(6):3292‐3302. doi:10.1111/jcmm.15002 31970862 PMC7131911

[138] Xia X , Xu Q , Liu M , et al. Deubiquitination of CD36 by UCHL1 promotes foam cell formation. Cell Death Dis. 2020;11(8):636. doi:10.1038/s41419-020-02888-x 32801299 PMC7429868

[139] Ji R , Gu Y , Zhang J , et al. TRIM7 promotes proliferation and migration of vascular smooth muscle cells in atherosclerosis through activating c‐Jun/AP‐1. IUBMB Life. 2020;72(2):247‐258. doi:10.1002/iub.2181 31625258

[140] Burger F , Baptista D , Roth A , Brandt KJ , Miteva K . The E3 ubiquitin ligase peli1 deficiency promotes atherosclerosis progression. Cells. 2022;11(13):11132014. doi:10.3390/cells11132014 PMC9265341 35805095

[141] Soehnlein O , Libby P . Targeting inflammation in atherosclerosis—from experimental insights to the clinic. Nat Rev Drug Discovery. 2021;20(8):589‐610. doi:10.1038/s41573-021-00198-1 33976384 PMC8112476

[142] Roy P , Orecchioni M , Ley K . How the immune system shapes atherosclerosis: roles of innate and adaptive immunity. Nat Rev Immunol. 2022;22(4):251‐265. doi:10.1038/s41577-021-00584-1 34389841 PMC10111155

[143] Wolfrum S , Teupser D , Tan M , Chen KY , Breslow JL . The protective effect of A20 on atherosclerosis in apolipoprotein E‐deficient mice is associated with reduced expression of NF‐kappaB target genes. Proc Natl Acad Sci U S A. 2007;104(47):18601‐18606. doi:10.1073/pnas.0709011104 18006655 PMC2141823

[144] Zhu C , Chen W , Cui H , et al. TRIM64 promotes ox‐LDL‐induced foam cell formation, pyroptosis, and inflammation in THP‐1‐derived macrophages by activating a feedback loop with NF‐kappaB via IkappaBalpha ubiquitination. Cell Biol Toxicol. 2023;39(3):607‐620. doi:10.1007/s10565-022-09768-4 36229750 PMC10406714

[145] Wang C , Xu W , Chao Y , Liang M , Zhang F , Huang K . E3 ligase FBXW2 is a new therapeutic target in obesity and atherosclerosis. Adv Sci. 2020;7(20):2001800. doi:10.1002/advs.202001800 PMC7578860 33101872

[146] Chandra D , Londino J , Alexander S , et al. The SCFFBXO3 ubiquitin E3 ligase regulates inflammation in atherosclerosis. J Mol Cell Cardiol. 2019;126:50‐59. doi:10.1016/j.yjmcc.2018.11.006 30448480 PMC7425077

[147] Liu M , Yan M , Lv H , et al. Macrophage K63‐linked ubiquitination of YAP promotes its nuclear localization and exacerbates atherosclerosis. Cell Rep.2020;32(5):107990. doi:10.1016/j.celrep.2020.107990 32755583

[148] Wertz IE , O'Rourke KM , Zhou H , et al. De‐ubiquitination and ubiquitin ligase domains of A20 downregulate NF‐kappaB signalling. Nature. 2004;430(7000):694‐699. doi:10.1038/nature02794 15258597

[149] Martens A , Priem D , Hoste E , et al. Two distinct ubiquitin‐binding motifs in A20 mediate its anti‐inflammatory and cell‐protective activities. Nat Immunol. 2020;21(4):381‐387. doi:10.1038/s41590-020-0621-9 32205881

[150] Kyaw T , Winship A , Tay C , et al. Cytotoxic and proinflammatory CD8+ T lymphocytes promote development of vulnerable atherosclerotic plaques in apoE‐deficient mice. Circulation. 2013;127(9):1028‐1039. doi:10.1161/CIRCULATIONAHA.112.001347 23395974

[151] Saigusa R , Winkels H , Ley K . T cell subsets and functions in atherosclerosis. Nat Rev Cardiol. 2020;17(7):387‐401. doi:10.1038/s41569-020-0352-5 32203286 PMC7872210

[152] Seijkens TTP , Poels K , Meiler S , et al. Deficiency of the T cell regulator Casitas B‐cell lymphoma‐B aggravates atherosclerosis by inducing CD8+ T cell‐mediated macrophage death. Eur Heart J. 2019;40(4):372‐382. doi:10.1093/eurheartj/ehy714 30452556 PMC6340101

[153] Jean‐Charles PY , Wu JH , Zhang L , et al. USP20 (ubiquitin‐specific protease 20) inhibits TNF (tumor necrosis factor)‐triggered smooth muscle cell inflammation and attenuates atherosclerosis. Arterioscler, Thromb, Vasc Biol. 2018;38(10):2295‐2305. doi:10.1161/ATVBAHA.118.311071 30354204 PMC6205742

[154] Min JW , Lu L , Freeling JL , Martin DS , Wang H . USP14 inhibitor attenuates cerebral ischemia/reperfusion‐induced neuronal injury in mice. J Neurochem. 2017;140(5):826‐833. doi:10.1111/jnc.13941 28029679 PMC5527549

[155] Hou W , Yao J , Liu J , et al. USP14 inhibition promotes recovery by protecting BBB integrity and attenuating neuroinflammation in MCAO mice. CNS Neurosci Ther. 2023;29(11):3612‐3623. doi:10.1111/cns.14292 37269080 PMC10580339

[156] Lange SM , Armstrong LA , Kulathu Y . Deubiquitinases: from mechanisms to their inhibition by small molecules. Mol Cell. 2022;82(1):15‐29. doi:10.1016/j.molcel.2021.10.027 34813758

[157] Chan WC , Liu X , Magin RS , et al. Accelerating inhibitor discovery for deubiquitinating enzymes. Nat Commun. 2023;14(1):686. doi:10.1038/s41467-023-36246-0 36754960 PMC9908924

[158] Ren J , Yu P , Liu S , et al. Deubiquitylating Enzymes in Cancer and Immunity. Adv Sci. 2023;10(36):e2303807. doi:10.1002/advs.202303807 PMC10754134 37888853

[159] Pabon NA , Zhang Q , Cruz JA , Schipper DL , Camacho CJ , Lee REC . A network‐centric approach to drugging TNF‐induced NF‐kappaB signaling. Nat Commun. 2019;10(1):860. doi:10.1038/s41467-019-08802-0 30808860 PMC6391473

[160] Farrell K , Jarome TJ . Is PROTAC technology really a game changer for central nervous system drug discovery? Expert Opin Drug Discovery. 2021;16(8):833‐840. doi:10.1080/17460441.2021.1915979 PMC8298267 33870803

[161] Bekes M , Langley DR , Crews CM . PROTAC targeted protein degraders: the past is prologue. Nat Rev Drug Discovery. 2022;21(3):181‐200. doi:10.1038/s41573-021-00371-6 35042991 PMC8765495

[162] Henning NJ , Boike L , Spradlin JN , et al. Deubiquitinase‐targeting chimeras for targeted protein stabilization. Nat Chem Biol. 2022;18(4):412‐421. doi:10.1038/s41589-022-00971-2 35210618 PMC10125259

[163] Virani SS , Alonso A , Aparicio HJ , et al. Heart disease and stroke statistics‐2021 update: A report from the American Heart Association. Circulation. 2021;143(8):e254‐e743. doi:10.1161/CIR.0000000000000950 33501848 PMC13036842

